# Edaravone dexborneol attenuates oxidative stress in experimental subarachnoid hemorrhage via Keap1/Nrf2 signaling pathway

**DOI:** 10.3389/fphar.2024.1342226

**Published:** 2024-05-30

**Authors:** Kunyuan Zhu, Shijun Bi, Zechao Zhu, Wenxu Zhang, Xinyu Yang, Jiashuo Li, Guobiao Liang, Chunyong Yu, Pengyu Pan

**Affiliations:** ^1^ Department of Neurosurgery, General Hospital of Northern Theater Command, Shenyang, China; ^2^ China Medical University, Shenyang, Liaoning, China

**Keywords:** subarachnoid hemorrhage, early brain injury, edaravone dexborneol, oxidative stress, KEAP1, Nrf2

## Abstract

**Background:**

Subarachnoid hemorrhage (SAH) serves as a disease characterized by high incidence rate, which is exceedingly prevalent and severe. Presently, there is no unambiguous or efficacious intervention for the neurological impairment following SAH. Administering multi-targeted neuroprotective agents to reduce oxidative stress (OS) and neuroinflammation caused by early brain injury (EBI) has been demonstrated to improve neurological function and prognosis following SAH. Edaravone dexborneol (EDB), a novel multi targeted neuroprotective medication, combines four parts edaravone (EDA) with 1 part (+)-borneol in proportion. Clinical trials conducted in China have revealed during 2 days of acute ischemic stroke (AIS), early administration of EDB leads to improved therapeutic outcomes compared to treatment in EDA monotherapy. Currently, there is no clear evidence that EDB can effectively treat SAH, therefore, our study aims to investigate its potential therapeutic effects and mechanisms on EBI after SAH.

**Method:**

We used the intravascular threading method to establish a mouse model of SAH to explore whether EDA and EDB could produce anti-OS and anti-apoptosis effects. Behavioral assessment of mice was conducted using the balance beam experiment and the modified Garcia scoring system. Neuronal damage due to OS and Keap1/Nrf2 signaling pathway were detected through techniques of immunofluorescence, Western blotting, spectrophotometry. The group of EDA and EDB were injected intraperitoneally for 72 h after SAH.

**Results:**

The experiment results indicated that EDB lead to remarkably positive results by significantly enhancing neurological function, reducing blood-brain barrier (BBB) injury, and effectively inhibiting neuronal apoptosis after SAH. Further examination indicated EDB significantly reduced the expression of Keap1 and increased the expression of Nrf2, and it inhibited MDA, and enhanced SOD activity after SAH. These outcomes surpassed the effectiveness observed in EDA monotherapy. However, the application of ML385 reversed the anti-OS effects of EDB and EDA.

**Conclusion:**

Our experimental findings indicated that EDB could activate Keap1/Nrf2 signaling pathway to reduce OS damage, thereby protecting neurological function and enhancing behavioral abilities after SAH. These outcomes could facilitate the creation of new approaches for the clinical management of SAH.

## 1 Introduction

Subarachnoid hemorrhage (SAH) is a severe neurological disorder, which result in substantial neurological impairment and potentially life-threatening complications (Claassen and Park, 2022). The primary cause is the intracranial aneurysm rupture, accounting for approximately 85% (Claassen and Park, 2022). Patients with SAH at 55 years averagely, experience a notable impact on their quality of life, health, and personal property safety (Macdonald and Schweizer, 2017). Neuronal damage after SAH can occur through various pathways, such as neuroinflammation and injury caused by oxidative stress (OS) (Xu W. et al., 2019; Zeng et al., 2021). Pathophysiological alterations, such as the disturbance of blood-brain barrier (BBB) or the occurrence of vasogenic and cytotoxic cerebral oedema, can contribute to early brain injury (EBI), ultimately resulting in deteriorating neurological impairment (Feng et al., 2022). At present, there is a lack of any recognized successful approach to tackle the neurological damage resulting from SAH. Therefore, we urgently need a new treatment with good curative effect. Research has demonstrated that multi-target neuroprotective agents, which help to reduce OS and neuroinflammation induced by EBI, can be significant interventions to improve neurological impairment and prognosis following SAH (Feng et al., 2022). The main components of edaravone dexborneol (EDB) are edaravone (EDA) and dexborneol. It was considered to be a novel neuroprotective agent (Xu J. et al., 2019). Wherein, EDA serves as free radical scavenger, safeguarding neurons by sequestering reactive oxygen species (ROS) (Suh et al., 2019). Then, by preventing lipid peroxidation, DNA damage, and vascular endothelial damage, EDA aided in reducing neuronal injury resulting from SAH (Kikuchi et al., 2013; Wu S. et al., 2014). Furthermore, the administration of EDA markedly reduced neuronal apoptosis in cases of SAH (Chen et al., 2022). Dexborneol, the primary compound in (+)-borneol, is a bicyclic monoterpenoid that is highly soluble in lipids (Liu et al., 2021). It assisted in the absorption of other medications and helped preserve the integrity of the tight junction proteins and BBB (Liu et al., 2011; Dong et al., 2018). By enhancing the superoxide dismutase (SOD) activity, reducing the malondialdehyde (MDA) level, and effectively alleviating OS harm to the body, dexborneol demonstrates its antioxidant properties (Hur et al., 2013). Furthermore, it had the potential to decrease the synthesis or conveyance of proinflammatory cytokines such as interleukin-1β (IL-1β), cyclooxygenase-2 (COX-2), and tumor necrosis factor-α (TNF-α), thereby mitigating the risk of inflammation (Chen et al., 2019). Studies evaluating the effectiveness of EDB have shown improved neurological results in patients who received EDB during 2 days of acute ischemic stroke (AIS) onset, as opposed to those treated solely with EDA (Xu J. et al., 2019; Xu et al., 2021). EDB has proposed to offer protection from AIS through various pathways, including anti-oxidative, anti-inflammatory, and inhibition of apoptosis (Hu R. et al., 2022). Studies have demonstrated that the conventional OS pathway kelch-like ECH-associated protein 1 (Keap1)/nuclear factor erythroid 2-related factor 2 (Nrf2) signaling pathway is effective in treating cerebral infarction (Zhang et al., 2023). Keap1 and Nrf2 collaborate to regulate the antioxidant reaction, presenting a possible treatment approach for inflammatory diseases. The imbalance of Keap1/Nrf2 complex decreases antioxidants and may be related to the advancement of SAH (Tu et al., 2019). After SAH, a pathophysiological process of global cerebral ischemia occurs (Sehba et al., 2013). Pathophysiological process of global cerebral ischemia after SAH. Emerging evidence indicated that EDB could diminish OS and inflammation in a cerebral infarction model through Keap1/Nrf2 signaling pathway (Yang et al., 2022). However, the efficacy of EDB in treating brain injury caused by SAH has not been conclusively established. Our hypothesis is that EDB could reduce neurological injury induced by SAH by inhibiting OS via Keap1/Nrf2. In our experiment, we evaluated the therapeutic efficacy of EDB treating SAH and elucidated the mechanisms involved, to provide a new clinical strategy for treating SAH.

## 2 Materials and methods

### 2.1 Experimental animals

The research study underwent a thorough review and approved by the Ethics Committee of the General Hospital of the Northern Theater command. A total of 410 C57BL/6 mice (adult male, aged 8–10 weeks, weighing 22–30 g) were employed for this investigation. These animals were procured from the Laboratory Animal Centre of the General Hospital of the Northern Theater command. Our experimental animals were accommodated in a standard light environment (12 h of darkness/12 h of light) within an SPF-class laboratory animal facility, ensuring they had access to sufficient food and water.

### 2.2 Experimental design

This experimental design contained the three parts, with the following:

Experiment 1: To examine the effectiveness of EDB and determine the optimal dosage, we allocated randomly 100 C57BL/6 mice to seven groups, namely, sham, SAH, SAH + vehicle, SAH + EDB 1.25 mg/kg, SAH + EDB 12.5 mg/kg, SAH + EDB 125 mg/kg and SAH + EDA10 mg/kg group (12 mice in each group). Neurological deficits were evaluated in each group by means of the modified Garcia and balance beam tests following SAH induction, with a sample size of six mice per group. Additionally, brain water content (left, right cerebral hemispheres, cerebellum) was measured at 24 and 72 h post-SAH to evaluate the severity of cerebral oedema, also with six mice per group. According to the aforementioned findings, we have determined that the ideal dose of EDB is 12.5 mg/kg. Then we employed EDA with 10 mg/kg as a comparative standard in this investigation. This chosen dosage will be utilized in the subsequent experiments.

Experiment 2: To examine the neuroprotective role of EDB in the context of SAH, we allocated stochastically 120 C57BL/6 mice into five distinct groups: sham, SAH, SAH + vehicle, SAH + EDB and SAH + EDA group (21 mice in each group). After inducing SAH, we quantified the permeability of Evans blue dye in the right and left cerebral hemispheres to assess BBB integrity. This assessment was carried out on a subset of six mice from each group. In addition, the levels of apoptotic proteins and signaling pathway proteins (Keap1 and Nrf2) were analysis using Western blotting (*n* = 6). Immunofluorescence staining (*n* = 3) was utilized to examine the level of apoptotic neurons in the left cerebral hemisphere subsequent to SAH. Additionally, a subset of six mice from each group were utilized to measure the content of SOD and MDA, which provide insight into brain OS level.

Experiment 3: To explore the specific mechanism of action of EDB, we allocated 190 C57BL/6 mice into six groups randomly: sham, SAH + vehicle, SAH + vehicle + EDB, SAH + ML385+EDB, SAH + vehicle + EDA and SAH + ML385+EDA group (27 mice in each group). After 24 h of inducing SAH, a subset of six mice from each group underwent several tests, included two behavioral experiment and measurements of brain water content (left, right cerebral hemisphere and cerebellum). The purpose was to evaluate neurological deficits and determine the severity of cerebral oedema. Western blotting analysis was undertaken on a subset of six mice from each group to identify apoptotic proteins and signaling pathway proteins. This analysis purpose was to examine the levels of these proteins and their potential contribution to the observed effects of EDB in relation to SAH. Immunofluorescence staining was carried out on a subset of three mice from each group to identify apoptotic neurons in the left cerebral hemisphere following SAH. Additionally, we measured SOD and MDA levels, providing insights into the degree of cerebral OS.

### 2.3 Drug doses

**FIGURE 1:**
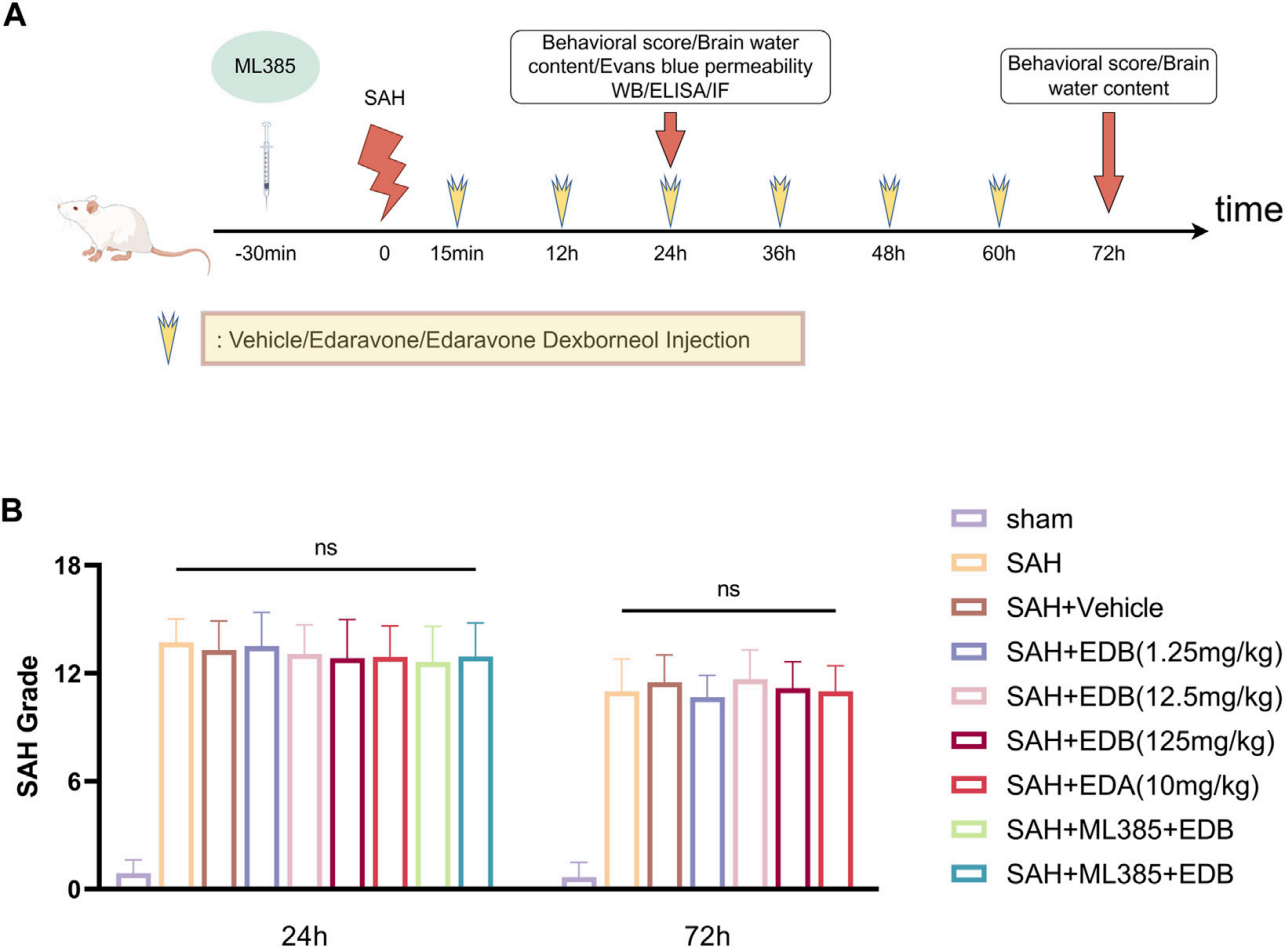
**(A)** Experimental flow chart; **(B)** SAH grading scores after SAH. No statistical difference in SAH grade indicated the SAH model are consistent and comparable in each group.

In our experiment, we administered intraperitoneal injections of EDB to mice at three different doses: a low dose of 1.25 mg/kg, a medium dose of 12.5 mg/kg, and a high dose of 125 mg/kg. The first injection was given 15 min after inducing SAH, followed by injections every 12 h until the mice died. These doses were determined according to previous studies on edaravone and our pre-experiments (Jangra et al., 2017; Herbet et al., 2019; Dang et al., 2022). On the other hand, the control group of mice received intraperitoneal injections of PBS buffer 15 min following SAH. In experiment 3, mice were intraperitoneally injected with ML385 (30 mg/kg, AbMole, Houston, United States) for 30 min before SAH based on previous studies (Singh et al., 2016; Hu Q. et al., 2022). EDA and EDB was administered after the administration of ML385 (Dang et al., 2022) (Figure 1A).

### 2.4 Mouse SAH model

This experimental study established the mouse SAH model on the left side using intravascular threading, as previously reported (Pan et al., 2017). Initially, mice were anaesthetized with 1% sodium pentobarbital (40 mg/kg) by intraperitoneal injection. Then the external carotid artery (ECA) was surgically separated using an operation microscope (Leica, Wetzlar, Germany), and a 2-mm incision was made at its distal end. Subsequently, insertion of a 5–0 nylon suture into the internal carotid artery (ICA) was performed through the previously made incision in the ECA until encountering a minimal degree of resistance. The suture was further advanced by an additional 2 mm to penetrate the site where the anterior cerebral and middle cerebral arteries bifurcate. Following the precise placement of the nylon suture at the intended location, it was cautiously withdrawn. To redirect blood flow from the common carotid artery (CCA) to the ICA and induce SAH, the ECA was securely ligated. The control group of mice underwent a similar procedure, nevertheless, the suture did not advance through the artery when encountering resistance. This procedure replicated the steps without inducing SAH.

### 2.5 Neurological function score

In the experiment, two assessment methods, namely, the modified Garcia score and the balance beam test, were utilized in a blinded method to assess the extent of neurobehavioral deficits post-SAH 24 and 72 h (Pan et al., 2020). The modified Garcia score was a comprehensive scoring system consisting of six tests, which were used to assess various aspects of neurological function. It allocated 18 points to judge the extent of neurological impairments. These tests assessed spontaneous activity, spontaneous limb movement, forepaw extension, climbing, body proprioception, and response to whisker stimulation. Each individual test in the modified Garcia score was scored from 3 to 18 points. Higher scores within this range indicate improved neurological function for that specific test. The balance beam test involved placing the mice on a beam, and their ability to walk along the beam within 1 minute was measured. The distance walked by the mice was then scored on a scale of 0–five points, with higher score indicating better motor coordination and balance.

### 2.6 SAH grading assessment

The experiment utilized an 18-point SAH severity grading system (Dong et al., 2016). The skull base of the mice was classified into six regions, and a grading system ranging from 0 to three was applied to assess the extent of bleeding observed in each region. A score of 0 indicated no hemorrhage in that region, while a score of one indicated a small amount of hemorrhage. A score of two denoted significant hemorrhage with clear vascular morphology, and a score of three indicated a large amount of hemorrhage with unclear vascular morphology. The total score of hemorrhage severity was calculated by summing up the scores of all six regions. Scores ranging from 0 to seven indicated mild hemorrhage, scores from 8 to 12 indicated moderate hemorrhage, and scores from 13 to 18 indicated severe hemorrhage. Experiment excluded mice with SAH scores below eight points.

### 2.7 Brain water content

The determination method of brain water content was the same as the previous experiment (Zou et al., 2021). Mice were euthanized by cervical dislocation method, then the brain was dissected into the left, right hemispheres, as well as the cerebellum. Subsequently, the tissue was subjected to a 105°C oven for a duration of 24 h in order to thoroughly eliminate all moisture. After the completion of the drying, the weight of the brains was once again measured in order to ascertain their dry weight. The formula for calculating brain water content was as follow:

$$\text{Brain water content}=\frac{\left(wet\;weight-dry\;weight\right)}{wet\;weight}\times 100\%$$

### 2.8 Evans blue penetration assessment

The BBB disruption was evaluated using Evans blue dye penetration as previously reported (Han et al., 2021). At post-24 h SAH, under 1% sodium pentobarbital anesthesia, we administered a 2% concentration of Evans blue dye (4 mL/kg) via the left femoral vein, followed by a 60-min circulation. In order to eliminate any residual dye in the vessels, mice were then perfused with PBS buffer. Brain tissue was collected and homogenized in a saline solution. Afterwards, the brain tissue that had been homogenized using frozen centrifuge (Thermo, Massachusetts, United States) was subjected to centrifugation at a speed of 15,000 revolutions per minute for a duration of 30 min. The collected liquid above the sediment was obtained, and an equivalent amount of trichloroacetic acid was introduced to cause protein precipitation. After being incubated overnight at 4°C, the mixture was subjected to another round of centrifugation at 15,000 rpm to collect the ultimate supernatant. The microplate reader (Epoch, Biotek, Winooski, United States) measurement of absorbance at 620 nm was conducted in order to determine the concentration of Evans blue dye present in the supernatant.

### 2.9 Western blotting

We extracted the mice brains and then proteins were then harvested from the left cerebral hemisphere and analyzed via Western blotting techniques as previously described (Li et al., 2020). Each well on the gel was supplemented with 40 μg of protein, followed by gel electrophoresis (Bio-rad, California, United States). The proteins were subsequently transferred onto a nitrocellulose membrane and sealed with a sealing buffer at ambient temperature for a duration of 60 min. After sealing, the nitrocellulose membrane was then subjected to incubation with a diluted primary antibody overnight at a temperature of 4°C. The main primary antibody included the antibodies of Keap1 (Proteintech, Wuhan, China), Nrf2 (Proteintech, Wuhan, China), β-tubulin (Proteintech, Wuhan, China), Bax (Santa Cruz, Texas, United States), Bcl-2 (Santa Cruz, Texas, United States), caspase-3 (Santa Cruz, Texas, United States), cleaved caspase-3 (Santa Cruz, Texas, United States), Parp1 (Proteintech, Wuhan, China) and Hsp90 (Bioss, Beijing, China). Subsequently, the membrane was incubated with an appropriately diluted secondary antibody for a period of 2 h at ambient temperature. The main secondary antibody included goat anti-rabbit IgG/HRP (Bioss, Beijing, China) and goat anti-mouse IgG/HRP (Bioss, Beijing, China). Chemiluminescence imaging system (Tanon 4,600, Tanon, Shanghai, China) was used to detect the bands which were then analyzed densitometrically by ImageJ.

### 2.10 Immunofluorescence staining

As previously, immunofluorescence staining was performed on fixed frozen section (Zhang et al., 2015). Following SAH, the mice were subjected to anesthesia using 1% sodium pentobarbital, followed by intracardial perfusion with a PBS buffer solution containing 4% PFA. Immediately euthanizing mice, and their brain tissues were gathered and immersed in 4% PFA for a duration of 24 h. Subsequently, the tissues were dehydrated in 30% sucrose for an additional 24 h. Afterwards, the specimens were quick-frozen at −35°C and coronal slices of the brain with a thickness of 8 µm were taken by using a freezing microtome (Leica, Wetzlar, Germany). To permeabilize, we used a solution of Triton X-100 at a concentration of 0.3% for 30 min at room temperature, after that, we sealed it with BSA for an additional 60 min at the same temperature. The sections were subsequently incubated with the NeuN antibody (Abcam, Cambridge, Britain) for an extended period of time at a temperature of 4°C. Following that, the sections were subjected to a fluorescent secondary antibody that corresponded to the source of the primary antibody, and this process was carried out at room temperature for a duration of 60 min. The main fluorescent secondary antibody included goat anti-mouse AF555 (Bioss, Beijing, China) and goat anti-rabbit AF555 (Bioss, Beijing, China). Next, Tunel Apoptosis Assay Kit (Beyotime, Shanghai, China) was done to detect apoptotic cells according to the manufacturer’s instructions. Lastly, the sections were sealed using the fluorescence quenching agent containing DAPI (Solarbio, Beijing, China). Finally, we observed these sections using the fluorescence microscope (BX-53, Olympus, Tokyo, Japan) to analyze the localization of the molecules.

### 2.11 Measurement of SOD and MDA

24 h after SAH, mice brain tissue was harvested. Subsequently, the brain tissues were homogenized in chilled PBS buffer at a ratio of 20 mg tissue per 100 µL buffer. Following the process of homogenization, we centrifuged and collected the supernatant. Finally, we utilized a microplate reader for the determination of SOD and MDA using Lipid Peroxidation MDA Assay Kit (Beyotime, Shanghai, China) and SOD Activity Assay Kit (Beyotime, Shanghai, China) according to the instructions (Han et al., 2022).

### 2.12 Statistics and analysis

Data were presented as mean ± SD (standard deviation). All of the data were tested for normality and variance homogeneity (Brown–Forsythe test). The outcomes between the two groups were compared with independent *t*-test, and Bonferroni corrected one-way ANOVA was chosen for contrast between multiple groups. The median (interquartile range) was used to describe the neurologic score results, and Mann-Whitney U test was carried out to compare differences. Statistical analysis was conducted using GraphPad Prism 9.0 and the statistical program SPSS 22.0, with statistical significance defined as *p* < 0.05.

## 3 Result

### 3.1 Mortality

None of the sham-operated mice died, eight mice died in SAH group, and 18 mice died in SAH + vehicle group, and nine mice died in SAH + EDA group, and 10 mice died in SAH + EDB group (3 mice in SAH + EDB 1.25 mg/kg group, five mice in SAH + EDB 12.5 mg/kg group, two mice in SAH + EDB 125 mg/kg group) and 14 mice died in SAH + ML385 group (7 mice in SAH + ML385 + EDA, seven mice in SAH + ML385+EDB) within 24 h after SAH due to severe hemorrhagic volume (Supplementary Figure S1). No mice were excluded from this study because of mild hemorrhagic volume without obvious neurological deficits (SAH grade score <8), and the SAH grade exhibited no statistical difference among each group (Figure 1B).

### 3.2 EDB improves neurological functions after SAH and alleviates BBB disruption

**FIGURE 2:**
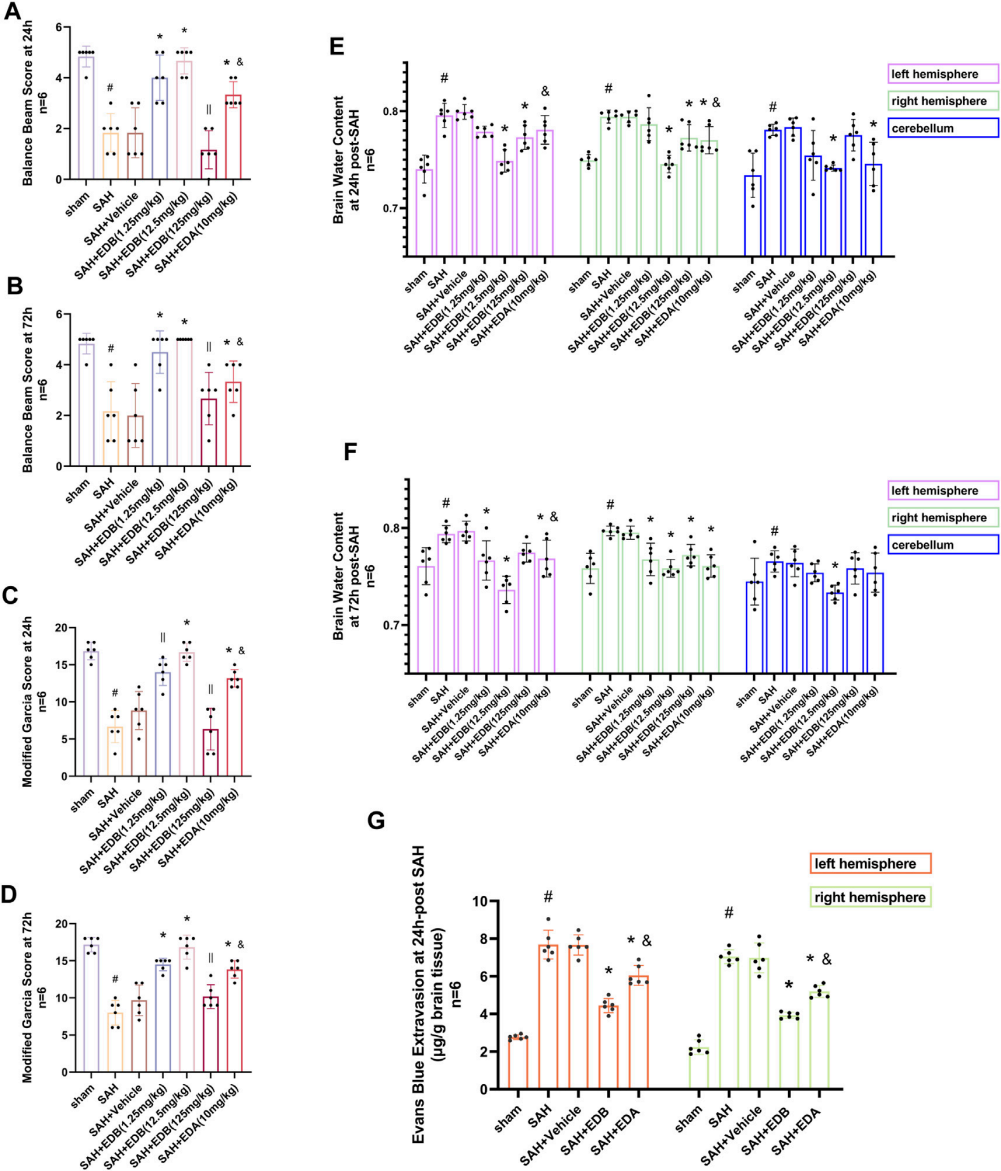
Neurological behavioral scores, brain water content of left, right hemisphere and cerebellum and Evans blue permeability after SAH. **(A and B)** Balance beam test scores at 24 and 72 h after SAH; **(C and D)** The modified Garcia scores at 24 and 72 h after SAH; **(E and F)** Brain water content at 24 h and 72 h post-SAH; **(G)** Evans blue permeability of left, right hemisphere at 24 h post-SAH. Data are presented as the mean ± SD. # *p* < 0.05 compared with sham; * *p* < 0.05 compared with SAH + vehicle; and *p* < 0.05 compared with SAH + EDB (12.5 mg/kg); || *p* ≥ 0.05 compared with SAH + vehicle.

For evaluating the EDB efficacy in treating neurobehavioral impairment, our experiment utilized a neurological function scoring system for the assessment of severity. At both 24 and 72 h following SAH, the SAH and SAH + vehicle groups exhibited notable neurological impairments in contrast to the EDB group, which demonstrated substantial enhancement in behavioral assessment, particularly in 12.5 mg/kg group (Figures 2A–D). After SAH, the group receiving EDB treatment demonstrated greater superiority compared to the group receiving only EDA treatment. Additionally, we also evaluated the impact of EDB on brain oedema after SAH through quantifying brain water content post-SAH. The findings indicated notable brain oedema in left, right hemispheres as well as in the cerebellum for both the SAH and SAH + vehicle group during the first 72 h after SAH. On the other hand, both the groups treated with EDA and EDB exhibited a notable alleviation of cerebral oedema. Furthermore, the treatment with a moderate dose (12.5 mg/kg) exhibited a markedly better outcome in comparison to the remaining two groups. In the left cerebral hemisphere at 24 h and 72 h after SAH, the therapeutic performances of EDA group and EDB group were in addition to significant differences, and the degree of cerebral oedema was reduced more significantly by EDB treatment (Figures 2E,F). Meanwhile, we also assessed the extent of BBB injury after SAH by measuring the permeability of Evans blue dye. The SAH and SAH + vehicle groups exhibited a significant leakage of Evans blue, suggesting a compromised integrity of the BBB after SAH. In comparison, both EDA and EDB treatments resulted in a significant reduction in Evans blue leakage, indicating attenuation of BBB damage (Figure 2G). Significantly, the effect with EDB treatment was significantly superior to the effect with EDA treatment, when compared to the EDA group. To summarize, in relation to enhancing neurobehavioral function, minimizing cerebral oedema, and reducing BBB disruption following SAH, the EDB treatment exhibited a notable therapeutic impact and outperformed the solitary use of EDA treatment. As a result of the experiment, 12.5 mg/kg EDB was found to be the optimal treatment dosage for SAH. Following a thorough analysis of the results, we opted for 12.5 mg/kg dosage of EDB for the forthcoming experiments.

### 3.3 EDB reduces neuro-apoptosis after SAH

**FIGURE 3:**
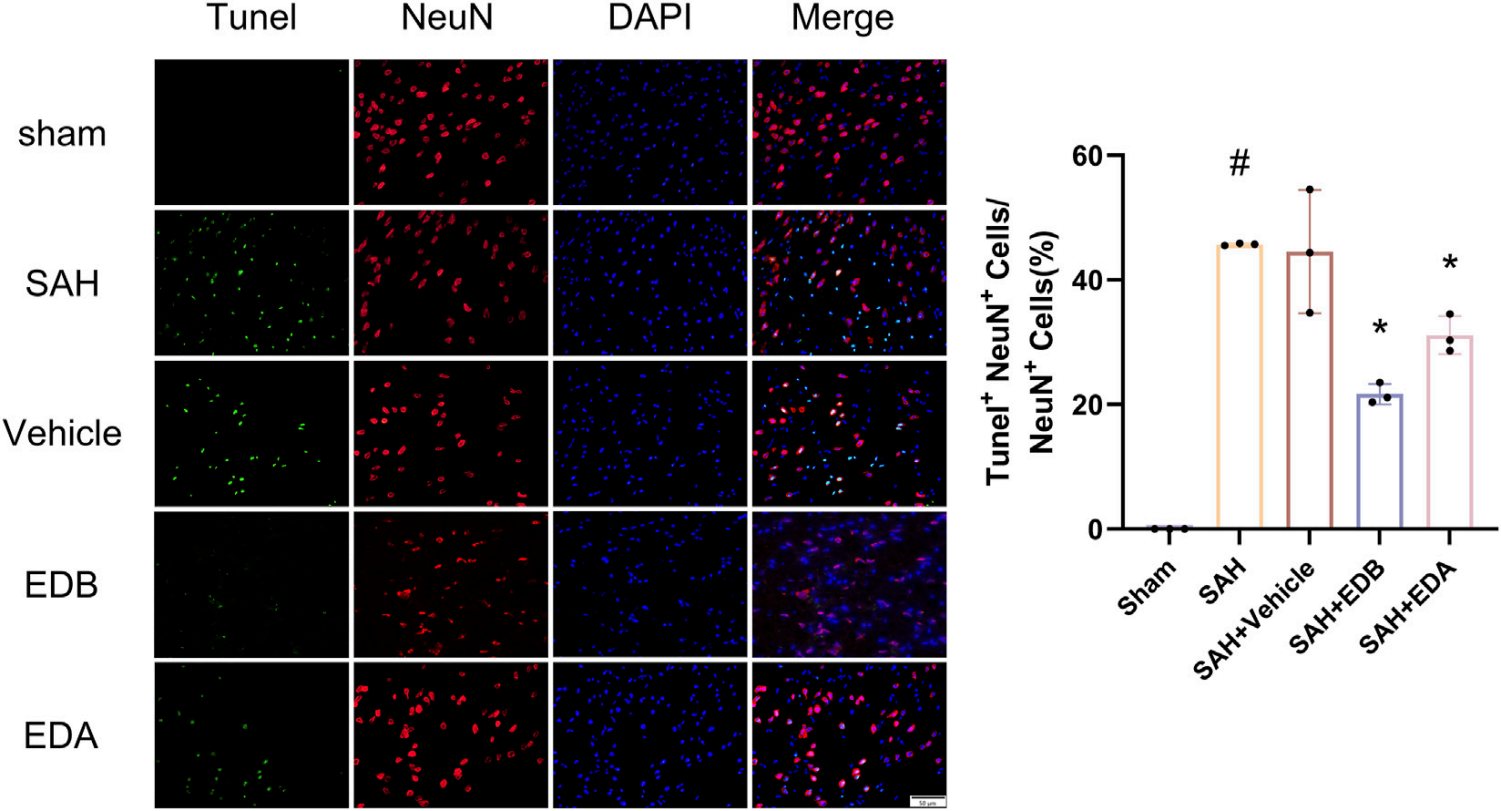
Neuronal Tunel immunofluorescence staining and ratio of Tunel-positive neurons to normal neurons. Data are presented as the mean ± SD. # *p* < 0.05 compared with sham; * *p* < 0.05 compared with SAH + vehicle.

**FIGURE 4:**
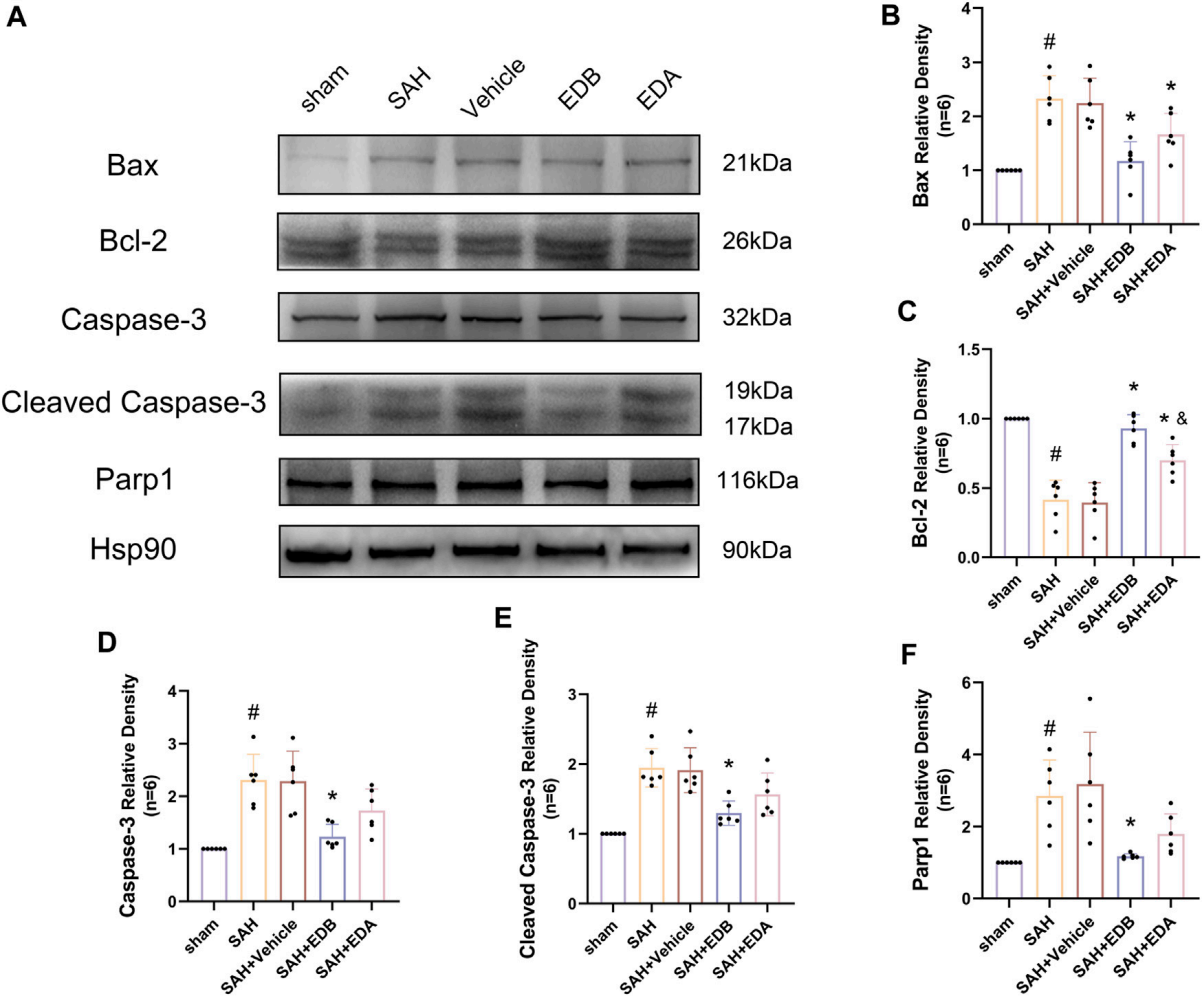
Western blotting and relative density analysis of apoptotic proteins. **(A)** The expressions of Bax, Bcl-2, caspase-3, cleaved caspase-3, Parp1 and Hsp90 were measured by Western blotting; **(B)** Bax relative density; **(C)** Bcl-2 relative density; **(D)** Caspase-3 relative density; **(E)** Cleaved caspase-3 relative density; **(F)** Parp1 relative density. Data are presented as the mean ± SD. # *p* < 0.05 compared with sham; * *p* < 0.05 compared with SAH + vehicle; and *p* < 0.05 compared with SAH + EDB.

To study the impact of EDB on decreasing neuronal apoptosis after SAH, our experiment employed immunofluorescence staining to assess the extent of neuronal apoptosis in brain tissue following SAH. The results obtained from both the SAH and SAH + vehicle groups demonstrated a significant elevation in the quantity of Tunel-positive neurons, suggesting a marked augmentation in neuronal apoptosis. Conversely, Tunel-positive neuron numbers in the EDA and EDB treatment groups exhibited a substantial decrease in comparison to the SAH + vehicle group, indicating the effectiveness of both EDA and EDB treatments in reducing apoptotic neurons after SAH (Figure 3). Furthermore, the EDB group exhibited a lower count of apoptotic neurons than the EDA group, suggesting the EDB treatment exhibited superiority over the EDA treatment alone. On the other hand, Western blotting was utilized to assess the levels of various proteins involved in the regulation of apoptosis. These proteins encompassed the pro-apoptotic proteins Bax, caspase3, cleaved caspase3 and Parp1, along with the anti-apoptotic protein Bcl-2 (Figure 4A). The results of the study revealed a notable reduction in Bcl-2 expression in SAH + vehicle group, while there was a noteworthy increase in the levels of Bax, caspase3, cleaved caspase3 and Parp1. However, the treatment of EDB effectively reversed these results. (Figures 4B–F). The findings demonstrated that EDB successfully decreased neural apoptosis following SAH. Moreover, the observed reduction in neuronal apoptosis after SAH exhibited a notably higher magnitude in the EDB treatment group in comparison to the EDA treatment group, suggesting EDB treatment was more effective than EDA treatment alone in reducing neuronal apoptosis.

### 3.4 EDB inhibits OS after SAH

**FIGURE 5:**
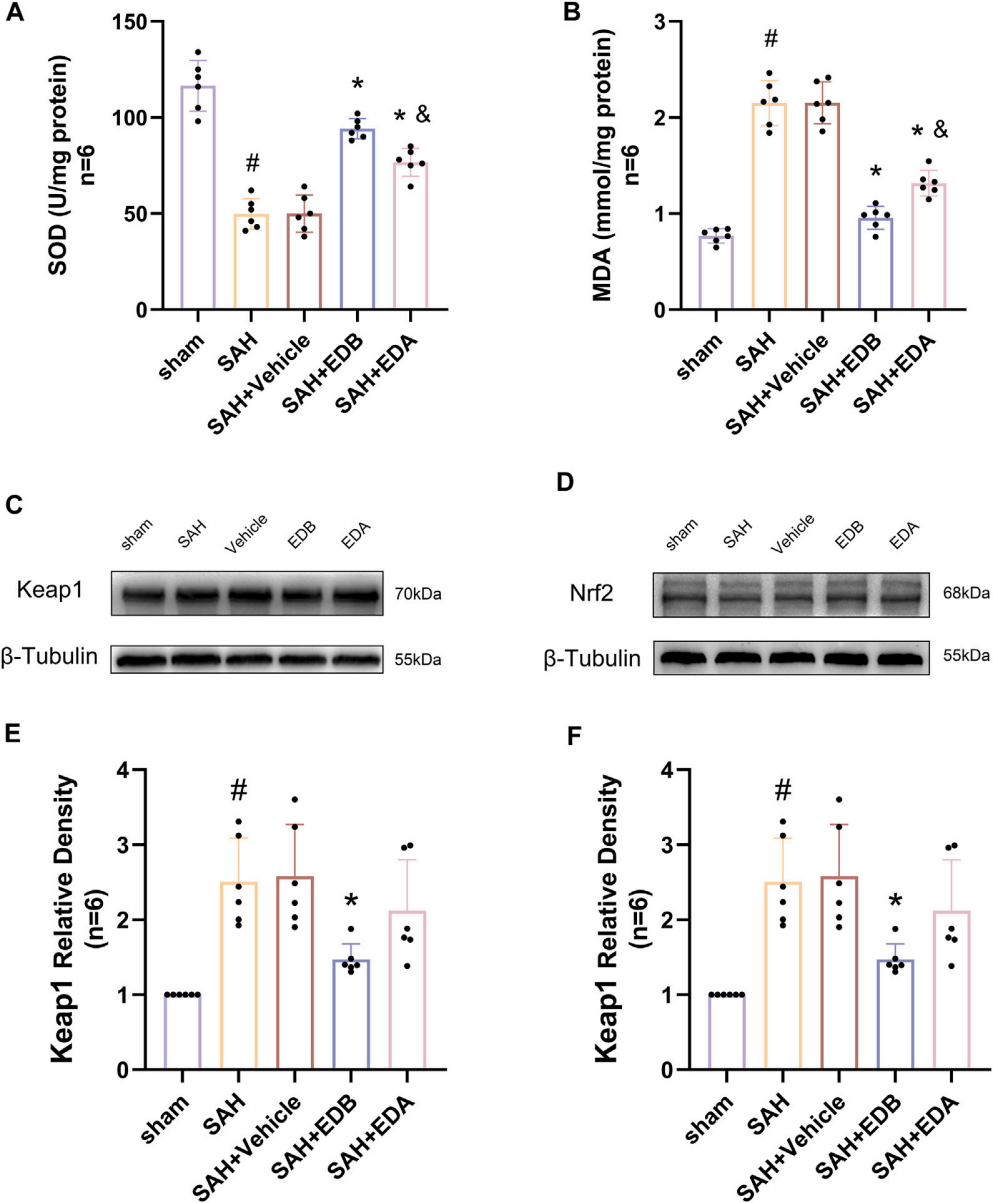
Measurement of oxidative stress indicators, Western blotting and relative density analysis of signaling pathway proteins. **(A)** The level of SOD; **(B)** The level of MDA; **(C)**The expressions of Keap1 were measured by Western blotting; **(D)** The expressions of Nrf2 were measured by Western blotting; **(E)** Keap1 relative density; **(F)** Nrf2 relative density. Data are presented as the mean ± SD. # *p* < 0.05 compared with sham; * *p* < 0.05 compared with SAH + vehicle; and *p* < 0.05 compared with SAH + EDB.

To investigate the impact of EDB therapy on the prevention of OS, we conducted an analysis of its antioxidant properties by assessing the levels of SOD and MDA at SAH post-24 h. The results indicated a noteworthy increase in MDA levels, as well as a notable decline in SOD levels in both SAH and SAH + vehicle groups, indicating heightened oxidative damage. In contrast to the control group, both the EDA and EDB treatment groups exhibited a reduction in MDA expression and an elevation in SOD levels (Figures 5A,B). Furthermore, the antioxidant impact of EDB was significantly greater in comparison to the sole administration of EDA. In conclusion, both EDA and EDB treatments exhibited remarkable antioxidant capacity against SAH-induced neural injury. Moreover, the antioxidant potential of EDB exceeded that of EDA treatment in isolation.

### 3.5 EDB attenuates OS injury after SAH via the Keap1/Nrf2 signal pathway

To identify the mechanisms by which EDB mitigates OS after SAH, we conducted Western blotting to assess the levels of Keap1 and Nrf2 (Figures 5C,D). There was a notable increase in Keap1 expression in both the SAH and SAH + vehicle groups, while Nrf2 expression decreased significantly. However, the groups treated with EDA and EDB demonstrated a noteworthy reduction in Keap1 expression and a notable elevation in Nrf2 expression when compared to the control group (Figures 5E,F). Moreover, EDB treatment group exhibited a more pronounced reduction in Keap1 expression and an elevation in Nrf2 expression in comparison to the group receiving EDA alone.

### 3.6 Blockade of Nrf2 can abolish the anti-OS of EDB

**FIGURE 6:**
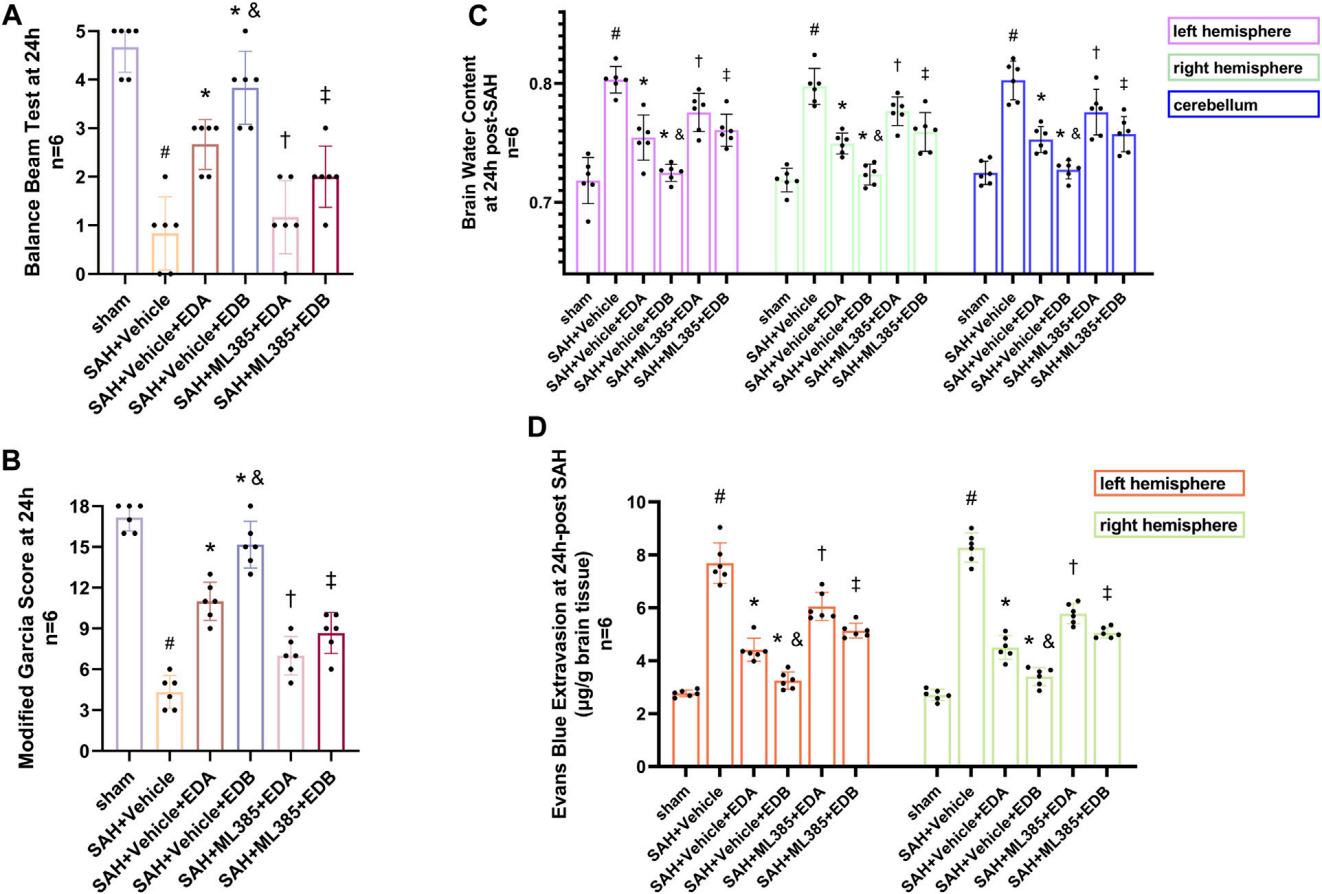
After application of the Nrf2 inhibitor (ML385) intervention, neurological behavioral scores, brain water content and Evans blue permeability after SAH. **(A)** Balance beam test scores at 24 h after SAH; **(B)** The modified Garcia scores at 24 h after SAH; **(C)** Brain water content of left, right hemisphere and cerebellum at 24 h after SAH; **(D)** Evans blue permeability after SAH. Data are presented as the mean ± SD. # *p* < 0.05 compared with sham; * *p* < 0.05 compared with SAH + vehicle; and *p* < 0.05 compared with SAH + EDA; † *p* < 0.05 compared with SAH + vehicle + EDA; ‡ *p* < 0.05 compared with SAH + vehicle + EDB.

**FIGURE 7:**
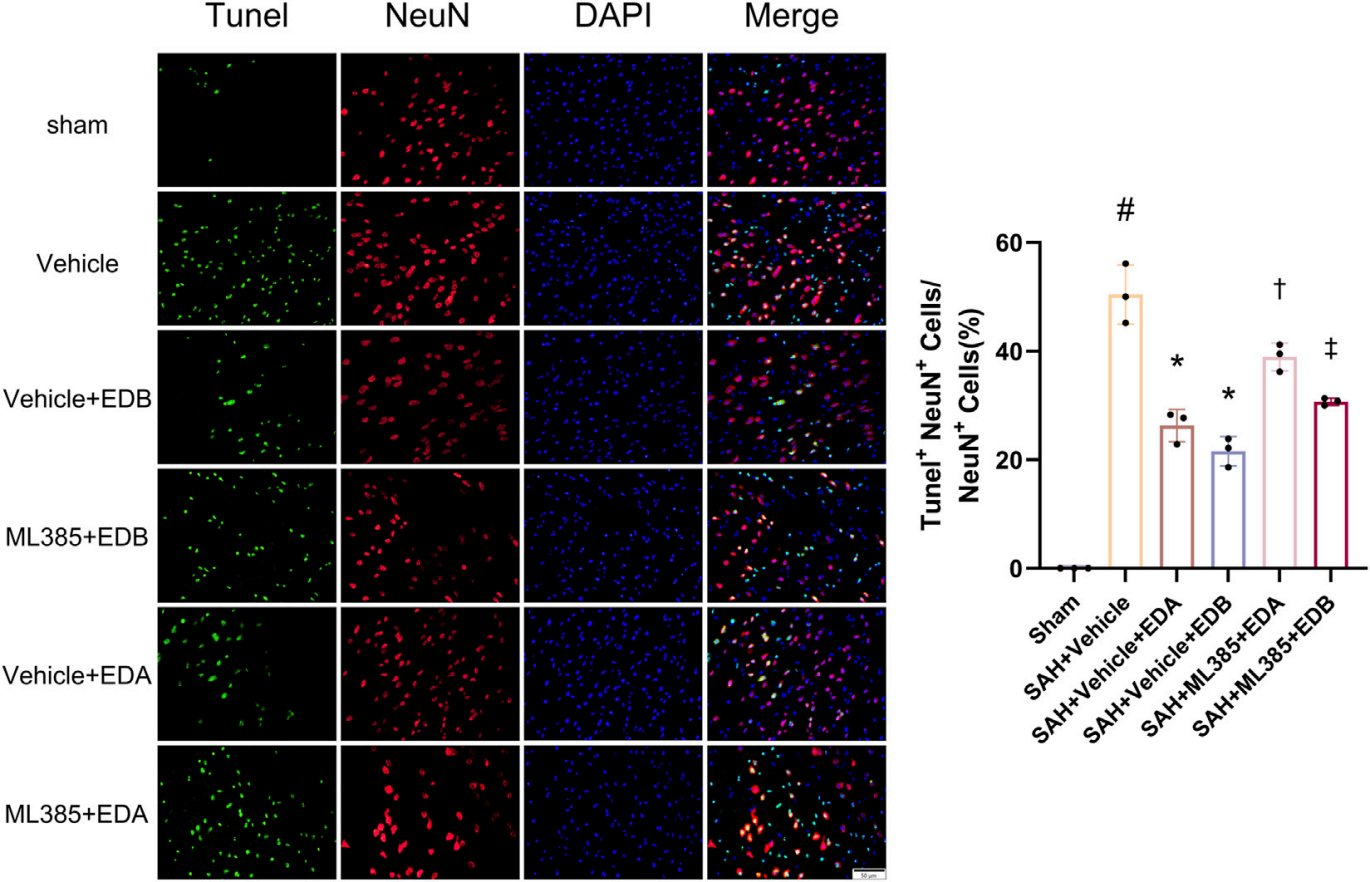
After application of the Nrf2 inhibitor (ML385) intervention, neuronal Tunel immunofluorescence staining and ratio of Tunel-positive neurons to normal neurons. Data are presented as the mean ± SD. # *p* < 0.05 compared with sham; * *p* < 0.05 compared with SAH + vehicle; † *p* < 0.05 compared with SAH + vehicle + EDA; ‡ *p* < 0.05 compared with SAH + vehicle + EDB.

**FIGURE 8:**
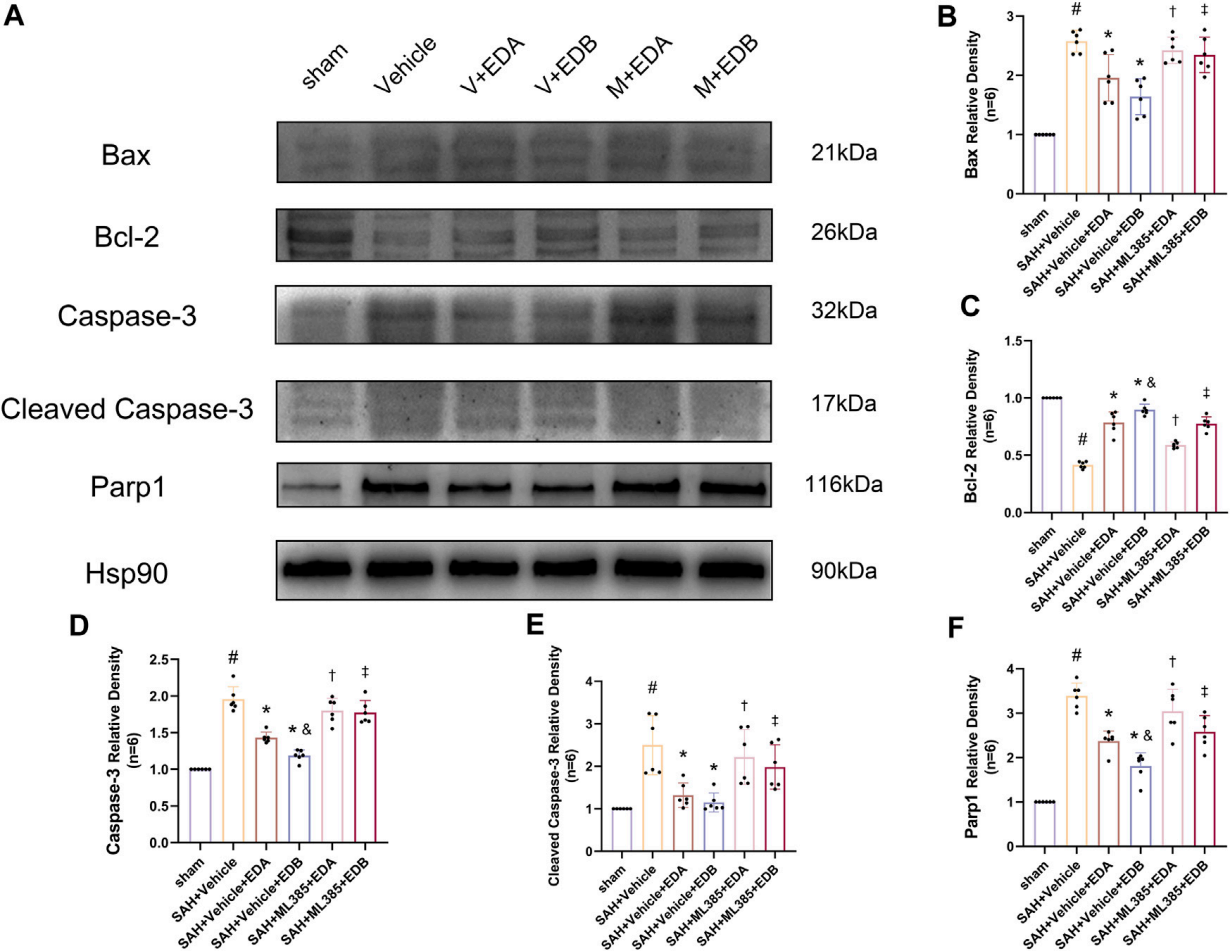
After application of the Nrf2 inhibitor (ML385) intervention, Western blotting and relative density analysis of apoptotic proteins. **(A)** The expressions of Bax, Bcl-2, caspase-3, cleaved caspase-3, Parp1 and Hsp90 were measured by Western blotting; **(B)** Bax relative density; **(C)** Bcl-2 relative density; **(D)** Caspase-3 relative density; **(E)** Cleaved caspase-3 relative density; **(F)** Parp1 relative density. Data are presented as the mean ± SD. # *p* < 0.05 compared with sham; * *p* < 0.05 compared with SAH + vehicle; and *p* < 0.05 compared with SAH + EDA; † *p* < 0.05 compared with SAH + vehicle + EDA; ‡ *p* < 0.05 compared with SAH + vehicle + EDB.

**FIGURE 9:**
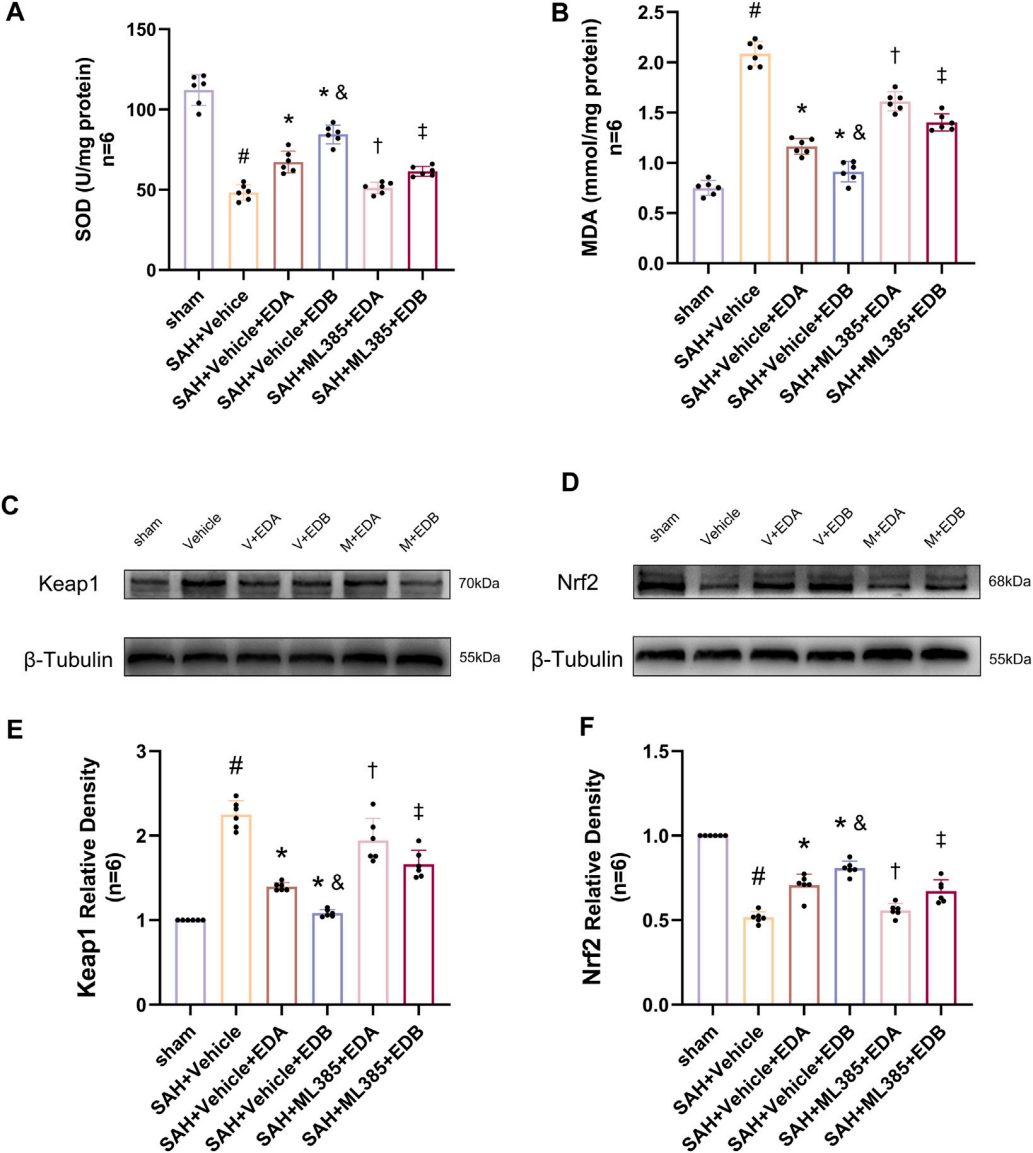
After application of the Nrf2 inhibitor (ML385) intervention, measurement of oxidative stress indicators, Western blotting and relative density analysis of signaling pathway proteins. **(A)** The level of SOD; **(B)** The level of MDA; **(C)**The expressions of Keap1 were measured by Western blotting; **(D)** The expressions of Nrf2 were measured by Western blotting; **(E)** Keap1 relative density; **(F)** Nrf2 relative density. Data are presented as the mean ± SD. # *p* < 0.05 compared with sham; * *p* < 0.05 compared with SAH + vehicle; and *p* < 0.05 compared with SAH + EDA; † *p* < 0.05 compared with SAH + vehicle + EDA; ‡ *p* < 0.05 compared with SAH + vehicle + EDB.

To further evaluate the effect of Keap1/Nrf2 signaling pathway on the antioxidant effect of EDB, the Nrf2 inhibitor ML385 was applied to SAH-induced mice at 30 min prior to drug administration. Compared with the SAH + vehicle group, two behavioral experiments score of the SAH + vehicle + EDA and SAH + vehicle + EDB groups showed significant improvement, whereas both ML385-treated groups showed poorer behavioral status; the behavioral scores of SAH + ML385+EDA were worse in contrast to the SAH + ML385+EDB group (Figures 6A,B). The findings of this study indicated that the inhibition of Nrf2 hindered the antioxidant properties of EDB in relation to the behavioral aspects of mice. The SAH + vehicle + EDA and SAH + vehicle + EDB groups exhibited a significant decrease in brain water content and Evans blue permeability (Figures 6C,D), whereas both groups showed a significant increase in these measures following ML385 treatment. These findings suggested that the ability of EDB to mitigate cerebral oedema and BBB disruption was significantly impeded when Nrf2 was blocked. To investigate the impact of ML385 on mitigating SAH-induced apoptosis following the administration of EDA and EDB, we analyzed the number of Tunel-positive neurons in the brain after SAH by immunofluorescence staining. The results showed that, compared with SAH + vehicle group, the number of Tunel-positive neurons decreased significantly. After the application of ML385, these results were reversed (Figure 7). On the other hand, Western blotting results showed the groups treated with SAH + vehicle + EDA and SAH + vehicle + EDB exhibited a noteworthy reduction in Bcl-2 expression and a notable increase in the expression of Bax, caspase3, cleaved caspase3 and Parp1 (Figures 8A–F). However, the administration of ML385 effectively reversed these results. The findings of this study demonstrated that when ML385 blocked the Keap1/Nrf2 signal pathway, the anti-apoptosis effects of EDA and EDB in reducing neuronal apoptosis were hindered. To detect the impact of ML385 on the reduction of SAH-mediated OS following the treatment of EDA and EDB, we quantitatively analyzed SOD and MDA in the brain tissue of mice after SAH. The results showed that the level of SOD increased and the level of MDA decreased significantly compared with SAH + vehicle group. Then after the application of ML385, these results were significantly reversed (Figures 9A,B). Meanwhile, we used Western blotting to detect the signaling pathway proteins Keap1 and Nrf2 (Figures 9C,D). In comparison to the SAH + vehicle + EDA and SAH + vehicle + EDB groups, the application of ML385 resulted in an elevation of Keap1 levels and a reduction in Nrf2 levels in both groups. These results demonstrated that ML385 could block the Keap1/Nrf2 signaling pathway to inhibit the anti-oxidative effects of EDA and EDB (Figures 9E,F).

## 4 Discussion

Our research findings have demonstrated the beneficial effects of EDB in ameliorating the neuronal detrimental consequences of OS, reducing neuronal apoptosis, and improving prognosis within the context of SAH. Additionally, we have provided evidence to substantiate the claim that the neuroprotective advantages of EDB in SAH are attained by means of activating anti-oxidative response by the Keap1/Nrf2 signaling pathway.

EDB serves as a new neuroprotective medication with multiple targets, blending EDA and dexborneol in a 4:1 ratio (Xu J. et al., 2019). Clinical trials evaluating the effectiveness of EDB have shown that patients who received EDB during 48 h of AIS had improved neurological status compared to those received EDA solely (Xu J. et al., 2019; Xu et al., 2021). Furthermore, the safety and tolerability with EDB at different doses were found to be more advantageous in comparison to the administration of EDA alone (Wu H. Y. et al., 2014). Research has shown that EDB for treating AIS has a neuroprotective mechanism that encompasses various pathways for neuroprotection, such as OS, apoptotic injury and inflammation response (Huang et al., 2022). Nevertheless, there is currently a lack of empirical evidence regarding the efficacy of EDB for treating SAH.

AIS patients have been treated with EDA, which acts as an antioxidant by scavenging free radicals (Rothstein, 2017). Numerous experimental studies suggest that EDA may reduce brain damage in SAH through multiple mechanisms. EDA is recognized for its antioxidant characteristics and its capacity to eliminate hydroxyl radicals, peroxyl radicals, and other ROS. This helps in decreasing OS and preventing the buildup of lipid peroxidation and oxidative DNA damage (Kikuchi et al., 2013; Wu S. et al., 2014; Suh et al., 2019). Multiple studies have provided evidence supporting the efficacy of EDA in SAH for the purpose of neuronal protection, neurological function enhancement, and mitigation of unfavorable prognostic outcomes subsequent to SAH (Yamashita and Abe, 2019). At the same time, we select EDA as a positive control drug to further explore the efficacy with EDB treatment.

Dexborneol, a bicyclic monoterpene compound, is the primary component of (+)-borneol. It assists in the uptake of other medications and protects the tight junction proteins and BBB. This protection helps prevent neurological harm in AIS (Liu et al., 2011; Dong et al., 2018; Liu et al., 2021). In addition, dexborneol has the ability to enhance SOD activity and decrease MDA levels, effectively diminishing OS harm within the body. It also suppresses the generation and manifestation of pro-inflammatory factors and associated proteins, thereby inhibiting the inflammatory response (Hur et al., 2013; Chen et al., 2019). The combination of EDA and dexborneol, known as EDB, has the potential to be more efficient compared to using EDA alone (Xu J. et al., 2019; Xu et al., 2021). Our current research examined the efficacy of SAH with EDB treatment and explored the distinctions compared to EDA therapy. The findings indicated that the utilization of EDB therapy following SAH exhibited superior effectiveness and neuroprotective benefits in comparison to EDA in isolation.

SAH serves as a type of cerebrovascular event caused by blood entering the subarachnoid space from a ruptured aneurysm. There are various causes for its occurrence, with the most frequent being ruptured cerebral aneurysms and traumatic hemorrhage (Claassen and Park, 2022). EBI is a common complication that typically happens within 72 h after SAH, it is responsible for the majority of fatalities and is the primary factor contributing to unfavorable outcomes after SAH (Rass and Helbok, 2019). Studies have identified OS as an important mechanism of EBI that leads to neurological dysfunction after SAH (Mo et al., 2019). OS is a physiological condition resulting from the elevation of ROS and free radicals, potentially resulting in cellular structural damage. Additionally, OS following SAH can result in neuronal apoptosis (Forman and Zhang, 2021; Wu et al., 2021).

**FIGURE 10:**
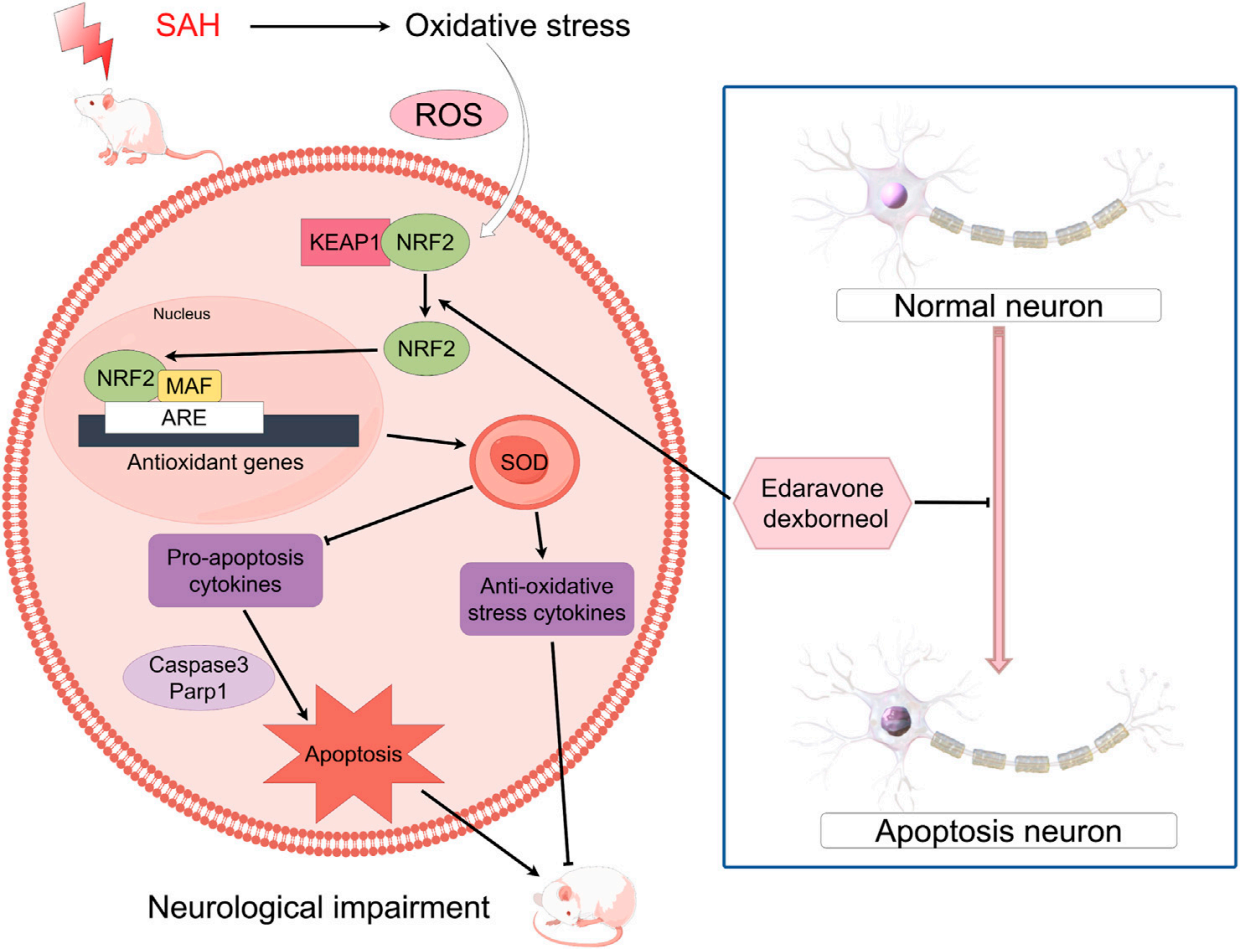
Mechanism of oxidative stress and neuronal apoptosis associated with EDB.

The Keap1/Nrf2 signaling pathway is of paramount importance in alleviating OS and exhibits a significant association with inflammatory disorders (Lu et al., 2016). Nrf2 is a prominent transcription factor implicated in OS transcription, constituting a key component within the repertoire of cell defense mechanisms (Liu et al., 2022). The role of Nrf2 is closely controlled by Keap1, a linker protein of the Cul3-based E3 ligase. In typical physiological circumstances, Keap1 engages with Nrf2 to direct the proteasome towards ubiquitin degradation (Baird and Yamamoto, 2020). When exposed to OS, Keap1 loses its activity and the process of Nrf2 ubiquitination stops (Yang et al., 2021). This leads to the buildup of newly produced Nrf2 and its subsequent activation. Consequently, Nrf2 is subsequently passed on to cell nucleus, initiating the transcription of numerous genes responsible for cellular defense, ultimately activating the defense mechanism (Adinolfi et al., 2023). In short, the Keap1/Nrf2 signaling pathway is of paramount importance in upholding cell redox equilibrium and dynamic homeostasis (Figure 10). Moreover, it actively supports anti-oxidative processes in SAH (Zhang et al., 2019). Our results showed EDA and EDB displayed strong antioxidant properties. In comparison to EDA alone, the use of EDB resulted in a greater decrease in OS-induced harm after SAH. Moreover, the combination of EDA and dexborneol exhibited increased neuroprotective properties against cell apoptosis following SAH.

The effectiveness of EDB in treating SAH was assessed in our experimental study. Moreover, we verified its ability to prevent OS and cell apoptosis while also uncovering the precise mechanism. In addition, there is also a need for research into the pivotal effect of EDB in other pathophysiological processes of SAH-mediated EBI, including neuroinflammation and vasospasm. Considering the extensive utilization of EDB in AIS, we eagerly anticipate forthcoming clinical studies involving individuals with SAH.

## 5 Conclusion

To summarize, this experiment demonstrates that EDB has a strong neuroprotective effect and improves behavioral function following SAH. The primary foundation of action appears to involve the initiation of the Keap1/Nrf2 signaling pathway, providing antioxidant activity as well as reducing neuronal apoptosis. These findings emphasize the potential for EDB as a neuroprotective agent with multi-target effects in mitigating neurological damage associated with SAH. The results of this study are of significant clinical importance and make a valuable addition to the advancement of novel SAH-treatment approaches.

## Data Availability

The original contributions presented in the study are included in the article/Supplementary Materials, further inquiries can be directed to the corresponding authors.

## Supplementary material

SUPPLEMENTARY FIGURE S1 Figure shows the percent survival of the study mice. None of the sham-operated mice died, 8 mice died in SAH group, and 18 mice died in SAH+ Vehicle group, and 9 mice died in SAH+ EDA group, and 10 mice died in SAH+ EDB group (3 mice in SAH+ EDB 1.25 mg/kg group, 5 mice in SAH+ EDB 12.5 mg/kg group, 2 mice in SAH+ EDB 125 mg/kg group) and 14 mice died in SAH+ ML385 group (7 mice in SAH+ ML385+ EDA, 7 mice in SAH+ ML385+ EDB) within 24 h after SAH due to severe hemorrhagic volume. Survival rates were significantly lower in the SAH group than in the sham group at 24 and 72 hours after subarachnoid hemorrhage. It was able to improve after applying EDA and EDB treatment, and the improvement was more significant in EDB.

## Abbreviations

EDA, Edaravone; EDB, Edaravone dexborneol; SAH, Subarachnoid hemorrhage; OS, Oxidative stress; EBI, Early brain injury; AIS, Acute ischemic stroke; IAS, Intracranial aneurysms; BBB, Blood-brain barrier; ROS, Reactive oxygen species; MDA, Malondialdehyde; SOD, Superoxide dismutase; TNF-α, Tumor necrosis factor-α; IL-1β, Interleukin-1β; COX-2, Cyclooxygenase-2; Keap1, Kelch-like ECH-associated protein 1; Nrf2, Nuclear factor erythroid 2-related factor 2; ICA, Internal carotid artery; ECA, External carotid artery; CCA, Common carotid artery; ANOVA, One-way analysis of variance.
