# Supplementary figures and images for: Edaravone dexborneol attenuates oxidative stress in experimental subarachnoid hemorrhage via Keap1/Nrf2 signaling pathway
Source: Front Pharmacol. 2024 May 30;15:1342226. doi: 10.3389/fphar.2024.1342226 (PMC11169797; doi:10.3389/fphar.2024.1342226)

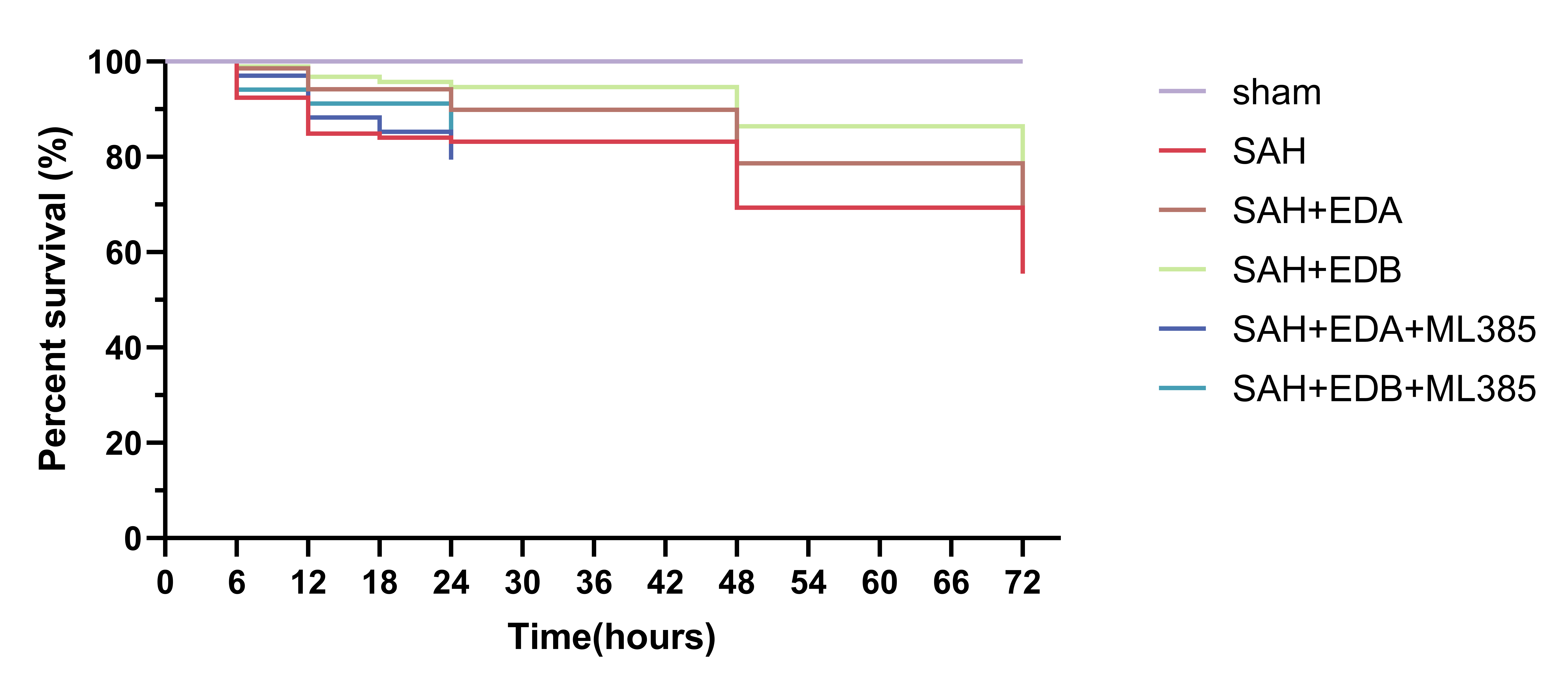

Supplement: Supplementary file 1 [file Image1.JPEG]
